# Parallels in Immunometabolic Adipose Tissue Dysfunction with Ageing and Obesity

**DOI:** 10.3389/fimmu.2018.00169

**Published:** 2018-02-09

**Authors:** William Trim, James E. Turner, Dylan Thompson

**Affiliations:** ^1^Department for Health, University of Bath, Bath, United Kingdom

**Keywords:** adipose, ageing, immunometabolism, obesity, inflammageing, immunosenescence

## Abstract

Ageing, like obesity, is often associated with alterations in metabolic and inflammatory processes resulting in morbidity from diseases characterised by poor metabolic control, insulin insensitivity, and inflammation. Ageing populations also exhibit a decline in immune competence referred to as immunosenescence, which contributes to, or might be driven by chronic, low-grade inflammation termed “inflammageing”. In recent years, animal and human studies have started to uncover a role for immune cells within the stromal fraction of adipose tissue in driving the health complications that come with obesity, but relatively little work has been conducted in the context of immunometabolic adipose function in ageing. It is now clear that aberrant immune function within adipose tissue in obesity—including an accumulation of pro-inflammatory immune cell populations—plays a major role in the development of systemic chronic, low-grade inflammation, and limiting the function of adipocytes leading to an impaired fat handling capacity. As a consequence, these changes increase the chance of multiorgan dysfunction and disease onset. Considering the important role of the immune system in obesity-associated metabolic and inflammatory diseases, it is critically important to further understand the interplay between immunological processes and adipose tissue function, establishing whether this interaction contributes to age-associated immunometabolic dysfunction and inflammation. Therefore, the aim of this article is to summarise how the interaction between adipose tissue and the immune system changes with ageing, likely contributing to the age-associated increase in inflammatory activity and loss of metabolic control. To understand the potential mechanisms involved, parallels will be drawn to the current knowledge derived from investigations in obesity. We also highlight gaps in research and propose potential future directions based on the current evidence.

## Introduction

Metabolically driven inflammation is a hallmark of cardiovascular disease and type-II diabetes mellitus (1). Several organs including the liver, the skeletal muscle, and the gut have a role in the generation and progression of these diseases. However, it has become clear that adipose tissue accumulation, sometimes within and around these organs, is a major driving force for metabolic and inflammatory dysfunction (1–3). Obesity, defined herein as a body mass index (BMI; ≥30 kg/m^2^), leads to extensive adipose tissue deposition and negative health consequences, due to chronic caloric overconsumption and an excessively sedentary lifestyle (4, 5). Globally, over 2.1 billion adults were overweight (BMI ≥ 25 to <30 kg/m^2^) or obese in 2014 (6). This number has been steadily increasing with global age-standardised mean BMI scores increasing by 0.4 and 0.5 kg/m^2^ each decade in men and women, respectively (7).

Obesity is characterised by poor metabolic control, oxidative stress, mitochondrial dysfunction, impaired immune function, and chronic, low-grade inflammation (8–13). In particular, it is well established that adipose tissue in obesity is a potent contributor to chronic, low-grade systemic inflammation, which can result in multiorgan dysfunction. One of the main factors associated with obesity-induced adipose tissue inflammation is dysregulation of the immune cell populations within the tissue stromal fraction, which contribute to the propagation of a pro-inflammatory microenvironment that spills over into the circulation and other organs (1, 14).

In contrast to obesity-centric research, the role of immunometabolic adipose tissue dysfunction in human ageing has been largely unexplored. In older people, many biological systems and processes become dysregulated (15). Indeed, age-associated immunological dysregulation potently modulates systemic inflammation, multiorgan dysfunction, and longevity (16–18). Moreover, molecular modulation of adipose tissue inflammatory and metabolic processes influences longevity in animal models, suggesting that adipose tissue may play an important role in the ageing process (19, 20). Further, in humans, an age-associated redistribution of adiposity towards the abdominal cavity occurs independently of obesity (21, 22). However, the role of adipose-resident immune cells and the interplay between metabolic and inflammatory processes in ageing adipose tissue is poorly characterised.

The aim of this review is to summarise the immunological alterations that take place in adipose tissue with obesity and ageing, with the aim to improve understanding of the role for adipose tissue immunometabolic dysfunction in age- and obesity-associated disease. This review first evaluates evidence showing obesity-specific immunological alterations in adipose tissue, which will then be used as a basis for understanding the potential role of adipose tissue immunological alterations in the pathophysiology of age-associated diseases.

## The Immune System and Inflammation

The immune system protects the host against viruses, bacteria, fungi, parasites, and tumours through a complex integration of innate and adaptive defences. The innate immune system provides a first line of defence, comprising physical barriers such as the skin and chemical barriers on surfaces exposed to the environment, preventing pathogens entering the body. In addition, the innate immune system has cellular defences, including monocytes, macrophages, natural killer (NK) cells, mast cells, neutrophils, eosinophils, basophils, and dendritic cells. Some cells possess characteristics of both the innate and adaptive immune system. For example, the small population of innate lymphocytes that are cluster of differentiation (CD) 1d restricted referred to as invariant natural killer T cell (iNKT) and other T-cells that express NK cell-associated surface proteins (NKT-like cells). Professional antigen-presenting cells link the two arms of the immune system. For example, dendritic cells act as tissue sentinels and, upon antigen encounter, travel from peripheral tissues (e.g., places in contact with the external environment; like the digestive system and dermis) to the lymph nodes where they activate cells of the adaptive immune system: B-cells and T-cells (23, 24). T-cells comprise 60–75% of all lymphocytes and originate from the bone marrow, maturing in the thymus, and circulate in blood as either antigen-naive or antigen-experienced cells (25). B-cells comprise 5–15% of all lymphocytes and elicit their effector functions, including their memory response, *via* soluble immunoglobulins (Igs), which can neutralise toxins or flag pathogens and target cells for elimination by other cells of the immune system such as macrophages and NK-cells (24).

In response to injury or infection, a local immune response is initiated, characterised by swelling, heat, and pain. One of the first local changes is an increase in blood flow facilitating an influx of acute-phase reactants, such as C-reactive protein, and an accumulation of innate and then adaptive immune cells for pathogen elimination and tissue repair. However, alterations to the tissue microenvironment and local stimuli can result in uncontrolled inflammation. Such alterations to the anti-inflammatory or pro-inflammatory milieu can disrupt systemic homeostasis and metabolic demand, perpetuating the inflammatory response that has profound health implications. A degree of inflammation within adipose tissue is central to tissue remodelling, as many of the cells, cytokines, and pro-oxidants produced at normal levels, regulate tissue homeostasis (26). However, prolongation of this normally transient and well-controlled process drives chronic, low-grade systemic inflammation that is central to the impaired health with obesity and ageing.

## Adipose Tissue Inflammation and Metabolic Disease

Impairments in adipose tissue function associated with structural and functional changes to the tissue results in the propagation of abnormal and often pro-inflammatory secretory profiles from adipocytes and cells of the stromal fraction. This association was first understood when murine obesity was linked with increased production of the inflammatory, insulin desensitising cytokine: tumour necrosis factor-α (TNF-α) (27). In the context of obesity, adipose tissue dysfunction is promoted by a chronic positive energy imbalance. Similar metabolic impairments are also observed in other conditions characterised by adipose tissue dysfunction, including ageing and lipodystrophy. Consequently, the similarities between these conditions allow for comparisons to be made to better understand the processes involved (28–30).

To date, a variety of stimuli for immunometabolic deterioration within adipose tissue have been proposed. These include increased gut-derived antigens (e.g., lipopolysaccharide), stimulation of immune cells by dietary or endogenously derived lipids, adipocyte hypertrophy—leading to apoptosis, necrosis, fibrosis, and hypoxia—and adipocyte dysfunction from mechanical stress (31). Collectively, these alterations impact various aspects of adipose tissue function, including changes to local blood flow, which impairs the endocrine potential of the tissue; changes to the extracellular matrix, which instigates monocyte infiltration to manage tissue remodelling; and adoption of a pro-inflammatory and pro-oxidative microenvironment, which act to recruit immune cells driving their pro-inflammatory polarisation (32–35). Moreover, the dysfunction of preadipocytes (adipocyte stem cell precursors) induced by a pro-inflammatory and pro-oxidative microenvironment inhibits the healthy turnover of adipose tissue, potentiated by, and impacting upon, impaired endothelial function, which exacerbates local hypoxia (34–36). The net result of these disturbances is the aberrant secretion of adipokines, which, *via* paracrine and endocrine means, impact appetite, bone health, metabolic health, and systemic inflammation through the activation of pro-inflammatory signal cascades [i.e., nuclear factor κB (NFκB), NLR family pyrin domain containing 3 (NLRP-3), and *c-*Jun *N*-terminal kinases (JNKs) signalling] (1, 37). Adipose tissue immunometabolic dysfunction also impacts the ability of adipocytes to buffer lipids. This dysfunction leads to the shunting of lipids towards non-adipose tissues, including the liver, the skeletal muscle, and the heart, promoting tissue-specific insulin resistance and inflammation, triggering β-cell, and metabolic dysfunction (1, 38, 39).

## Methodological Considerations in Adipose Tissue Immunological Research

Adipose tissue is an immunometabolically active organ in a constant state of flux (40). Therefore, assessment of adipose tissue function is a difficult task, and the nature of sampling tissue means the available data tend to represent a snap-shot of adipose physiology and function. In the context of the current review, this means that the assessment of adipose cellular composition at a given point in time will not necessarily provide a full and complete picture of the events that have led to the characterised phenotype. In human research, adipose tissue samples are often collected under fasting conditions, and very little information is available in the post-prandial state where much of the day is spent, which potently alters adipose tissue immunometabolic status (41). Furthermore, in human studies, the adipose tissue sampling methodology (e.g., surgical versus aspiration biopsies) may influence some outcomes (42). In mice, both the breed and inconsistency in whether animals are fasted or fed has the potential to influence their metabolic profile (43). Some investigations have adopted arteriovenous difference blood sampling techniques to sample the blood flowing across the subcutaneous adipose depots in an attempt to understand the dynamic behaviour of the tissue (44). This methodology allows for a greater understanding of how adipose tissue responds, in real-time, potentially giving a more complete picture of adipose tissue functionality.

Adipose tissue is distributed throughout the body with site-specific physiological properties. In humans, abdominal subcutaneous and visceral adipose tissue differ substantially in function and cellularity, and considerable depot differences are also apparent between gluteofemoral and abdominal adipose tissues, which is particularly relevant when comparing males and females due to sex-specific adipose tissue distribution (45, 46). In mice, different adipose tissue depots also possess unique physiological properties both between and within specific depots. Although beyond the scope of this review, differences include adipocyte size and expandability in response to high-fat overfeeding, control of lipolysis, and fatty acid and leukocyte composition (47, 48). Most investigations of adipose tissue immunology have been conducted with animal models, with considerably less work with humans. Although differences in adipose tissue biology between humans and animals is beyond the scope of this review, it is important to recognise that human adipose tissue is, indeed, very different to that of animals such as mice in terms of functionality, physiology, distribution, and make-up (i.e., quantities of brown and white adipose tissue, respectively) (49).

**Figure 1:**
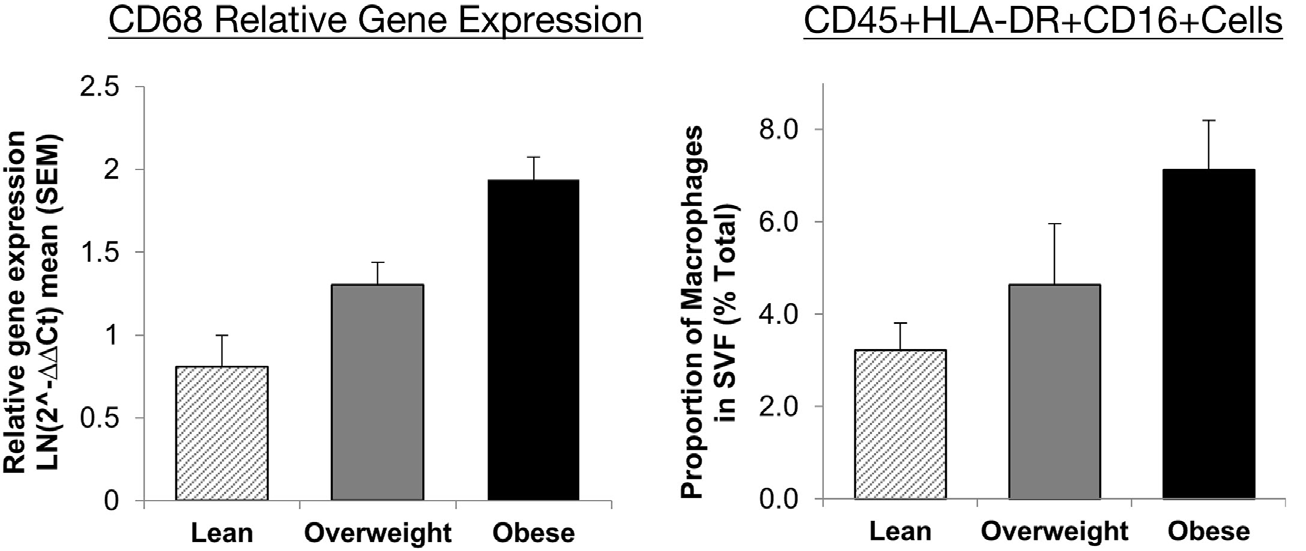
Agreement between RT-PCR and flow cytometric assessment of macrophage abundance in lean, overweight, and obese individuals subcutaneous abdominal adipose tissue. Relative gene expression of CD68 was used to identify macrophages (*n* = 30) and presented as mean 2^−ΔΔCt^ ± SEM. Proportions of macrophages in the adipose tissue SVF as a percentage of total cells (*n* = 17). Macrophages were identified as CD45+HLA-DR+CD16+ cells. Presented with permission from Travers et al. (35). Abbreviations: CD, cluster of differentiation; HLA-DR, human leukocyte antigen-antigen D related; RT-PCR, reverse transcription polymerase chain reaction; SVF, stromal vascular fraction.

To assess the constituents of the adipose tissue stromal fraction, many approaches have been employed, including immunohistochemistry, analysis of gene expression and protein level, and flow cytometry. Each method has benefits and drawbacks, which are summarised in Table 1. For example, it has been shown that immunohistochemistry yields reproducible results, but with flow cytometry, the recovery of macrophages after tissue digestion can be a possible source of variation (50). However, there is good agreement between flow cytometric analysis of adipose tissue cellular composition and the expression mRNA for key cell surface markers across a range of adiposities in humans (Figure 1) (35). Moreover, differences between studies in the selection of cell markers, coupled with lack of consensus over cell identification strategies, make direct comparison between studies complex and could feasibly explain conflicting results.

**Table 1:** Appraisal of three commonly utilised methods of assessing the cells of the adipose tissue stromal fraction.

Method	Benefits	Drawbacks
Immunohistochemistry	– Small amount of tissue required – Information on cell locality – Intact undisturbed tissue sample – Long-term storage – Some sample remains available for future analysis	– Manual counting is subject to investigator bias – Time consuming for manual counting – Limited to 2 or 3 antibodies per tissue slice

Flow cytometry	– Commonly 10–12 antibodies can be used in parallel – Thousands of cells analysed from one sample – Absolute cell counts can be determined – Isotype control and fluorescence minus one tubes can be added to an experiment to minimise the influence of subjectivity when gating populations	– Extensive processing required to extract the stromal fraction possibly leading to artefacts – Possibility for selective cell type loss with processing – Cell autofluorescence complicates gating of some populations (i.e., macrophages) – Large (>300 mg) tissue sample required – Small margin for bias when gating cell populations

Gene and protein expression (RT-PCR and Western blot)	– Very little tissue required – Little processing of the tissue required prior to freezing – Long-term storage – Some sample remains available for future analysis	– Requires isolation of a specific marker not expressed on any other cell type – Cannot be multiplexed by examining multiple markers on cell types

*RT-PCR, reverse transcription polymerase chain reaction*.

The mode of reporting assessments of cellular composition also requires consideration. Reporting proportional representation of cell types within the stromal fraction versus absolute cell counts can produce different findings. For instance, a proportional decline in certain populations in relation to total stromal cell yields may be a consequence of an expansion of another cell population within the tissue instead of an absolute decline in specific cell numbers. For example, one report showed that, compared to older mice, young mice exhibited around twice as many macrophages in adipose tissue when expressed as a percentage of the total stromal yield. However, these age-related differences were not seen when data were expressed per gram of adipose tissue (51). In summary, when interpreting the results of different studies, it must be considered whether the laboratory methods and data presentation/expression could in-part explain discrepant results, especially when interpreting cell counts and/or proportions within adipose tissue.

## Traditional Concepts in Adipose Tissue Immunology: An Inflammatory Role for Monocytes and Macrophages

Macrophages were first discovered within adipose tissue forming multinucleated crown-like structures of ~15 cells clustering around dead or dying adipocytes in obese C57/BL6J mice (52). In young obese mice and humans, these structures have been shown to comprise 90% of all adipose tissue macrophages (53). Macrophages are the main leukocyte in the adipose stromal fraction of young, non-obese mice and humans, comprising 4–15% of total stromal cell count (54, 55). Macrophage content increases with adiposity, and the accumulation is greatest in visceral depots in humans (55, 56). Adipose tissue macrophages are derived from monocytes, which differentiate in response to growth factors, including macrophage colony-stimulating factor and granulocyte–macrophage colony-stimulating factor (57, 58). Monocytes, defined by their cell surface expression of CD14 and CD16 (see Table 2), circulate in peripheral blood until they migrate into adipose tissue primarily in response to chemokine (C-C motif) ligand-2 (CCL-2)—also known as monocyte chemoattractant protein 1 (MCP-1)—released from adipose tissue (57, 59). MCP-1 promotes the local proliferation of adipose-resident macrophages, contributing to the large accumulation of these cells with obesity (60). Monocytes also enter the adipose microcirculation in response to adipocyte-derived cell-stress markers, including CCL-5, also known as regulated on activation, normal T-cell expressed and secreted (RANTES), interleukin (IL)-6, interferon-γ (IFN-γ), and TNF-α (61). Production of these signals, which enhances macrophage accumulation, also polarises macrophages into highly inflammatory, classically activated cells, referred to herein as M1-like macrophages to be consistent with the reporting of data in the studies we summarise. However, we acknowledge that the M1 and M2 classification of macrophages is simplistic and becoming outdated^1^ (see Table 2).

**Table 2:** Adipose tissue macrophage phenotypic classifications in humans.

Type	Cell surface expression profile	Secretion profile	Function
M1-like *“classically activated”*	CD14+CD16++ CD11c+CD31+CD44+CD45+CD206−HLA-DR+	TNF-α, IL-6, IL-8, IL-1β, NO	Pro-inflammatory, pro-oxidative, antimicrobial, pathogen clearance
Intermediate population	CD14+CD16+ CD11c+CD45+CD206+	TNF-α, IL-6, IL-8	Pro-inflammatory and pro-oxidative, tissue remodelling
M2-like *“alternatively activated”*	CD14++ CD16+ CD45+CD163+CD206+	IL-10, TGF-β	Anti-inflammatory, fibrosis, tissue surveillance, angiogenesis

*Text in italics and quotes emphasises other common terms to describe these cells. Intermediate population refers to adipose tissue macrophages expressing M2-like surface markers (e.g., CD206), while demonstrating an M1-like pro-inflammatory function. Expression pattern of CD14 and CD16 is best established on monocytes rather than macrophages. It is important to note that macrophage populations within adipose tissue can express markers of alternatively activated cells (e.g., CD206+) while also imparting a pro-inflammatory, classically activated function within the tissue, highlighting the spectrum of macrophage polarity within adipose tissue*.

*CD, cluster of differentiation; HLA-DR, human leukocyte antigen-antigen D related; IL, interleukin; NO, nitric oxide; TGF-β, transforming growth factor-beta; TNF-α, tumour necrosis factor-α*.

It has been consistently shown that there is a positive relationship between the number of macrophages in adipose tissue and the degree of whole-body insulin resistance in mice and humans (35, 62, 63). Moreover, TNF-α—which is primarily produced by macrophages in adipose tissue—is a potent inhibitor of insulin signalling and an activator of NFκB signalling (27, 64). In obese and inflamed adipose tissue, NFκB is activated by the fatty acid chaperone—Fetuin-A—interacting with the lipid-sensing toll-like receptor 4 (TLR-4) on the macrophage cell surface (65–67). In the commonly studied 3T3-L1 adipocyte cell line, TNF-α has been shown to directly impact insulin signalling by inactivating insulin receptor substrate 1 (IRS-1) through p44/42 mitogen-activated protein kinase (MAPK) activation (68). Chronic activation of pro-inflammatory stress sensors and signalling pathways: p44/42, JNK, p38, and MAPK by TNF-α derived from subcutaneous adipose-resident macrophages indirectly stimulates lipolysis in adipocytes, impairing lipid handling capacity (69–71). It has also been observed, *in vitro*, that monocytes incubated with palmitate—an abundant saturated fatty acid—exhibit upregulated IL-6 and TNF-α mRNA (72). Further, upon macrophage–adipocyte co-culture, there is a substantial increase in macrophage-derived TNF-α and IL-1β (65). As TNF-α, IL-6, and IL-1β stimulate a feed-forward activation of NFκB and JNK, this leads to IRS-1 phosphorylation at serine residues rather than tyrosine, perpetuating insulin signalling impairments (73). Another factor influencing macrophage function in adipose tissue is hypoxia brought about by adipocyte expansion, without concurrent angiogenesis, impairing oxygen supply (74, 75). Hypoxia potentiates the palmitate-induced pro-inflammatory and pro-oxidative polarisation of human macrophages, while hypoxia-inducible factor-1α (HIF-1α) can directly induce pro-inflammatory M1-like macrophage polarisation and pro-inflammatory cytokine productions (76, 77). Macrophage-derived reactive oxygen species produced as a result of hypoxia may also impair lipid and glucose metabolic control through the S-nitrosylation of the peroxisome proliferator-activated receptor-γ (PPAR-γ) nuclear receptor (78).

Macrophages are considered central regulators of fibrosis and may also increase progenitor cell deposition into the extracellular matrix as a means of protecting hypertrophic adipocytes from rupturing (59). M2-like macrophages possess a pro-fibrotic potential and are, therefore, recruited into the adipose tissue in response to “danger signals” (i.e., cytokines, acute-phase proteins, and stress signal cascades) released from hypertrophic adipocytes (79). However, the exposure of macrophages to hormones, cytokines, chemokines, and fatty acids found within dysfunctional adipose tissue encourages them to adopt a pro-inflammatory phenotype potentiating metabolic deteriorations and inflammation (80, 81). In human obesity, perhaps counter intuitively based on the simple macrophage phenotyping, it is the M2-like, and not M1-like, macrophages, with a tumour-associated genetic signature and remodelling phenotype that correlates with obesity in subcutaneous adipose tissue (33). Another stimulus that might influence macrophage infiltration into adipose tissue is endoplasmic reticulum stress, which promotes a pro-inflammatory microenvironment through the initiation of the unfolded protein response. In mice, increased fatty acid-mediated oxidative stress upregulates pro-inflammatory cytokine expression, which, in humans, is also initiated by elevated TNF-α and IL-1β (82, 83). Endoplasmic reticulum stress responses involving the inositol-requiring enzyme 1α and CCAAT-enhancer-binding protein homologous protein direct pro-inflammatory, M1-like macrophage polarisation in adipose tissue (84, 85). Finally, it is unknown whether macrophages initiate inflammatory responses in adipose tissue or whether other cells present stimulate macrophage accumulation and dysfunction. For example, some studies in mice indicate that macrophages infiltrate adipose tissue in the absence of other leukocytes, whereas other studies show that different leukocyte subsets are central to macrophage accumulation (86).

Recent evidence has indicated that a unique adipose-resident macrophage population exists. In mice fed a high-fat diet, a population of sympathetic neuron-associated macrophages accumulate within visceral adipose tissue. This macrophage population regulates adipocyte exposure to norepinephrine by sequestering and degrading norepinephrine released into the adipose tissue interstitium. This process is brought about by the selective expression of the norepinephrine transporter, *SLC6A2*, and genes controlling norepinephrine-degradation such as monoamine oxidase-A by these macrophages (87). However, unlike other adipose-resident macrophage populations, these sympathetic neuron-associated macrophages do not increase within obese adipose tissue *via* proliferative mechanisms, but instead appear to infiltrate the tissue selectively (87). Given that catecholamines increase lipolytic rate in adipocytes *via* adrenergic receptors triggering the downstream hydrolysis of triglycerides, selective knockout of these sympathetic neuron-associated macrophages protects against high-fat diet-induced obesity, in mice. Moreover, the capacity to buffer regional norepinephrine releases, which in healthy adipose tissue may act as a protective mechanism to avoid the dangerous effects of chronic exposure to norepinephrine, is also sufficient to modulate overall sympathetic tone in murine adipose tissue, influencing whole-body metabolism (88).

**Figure 2:**
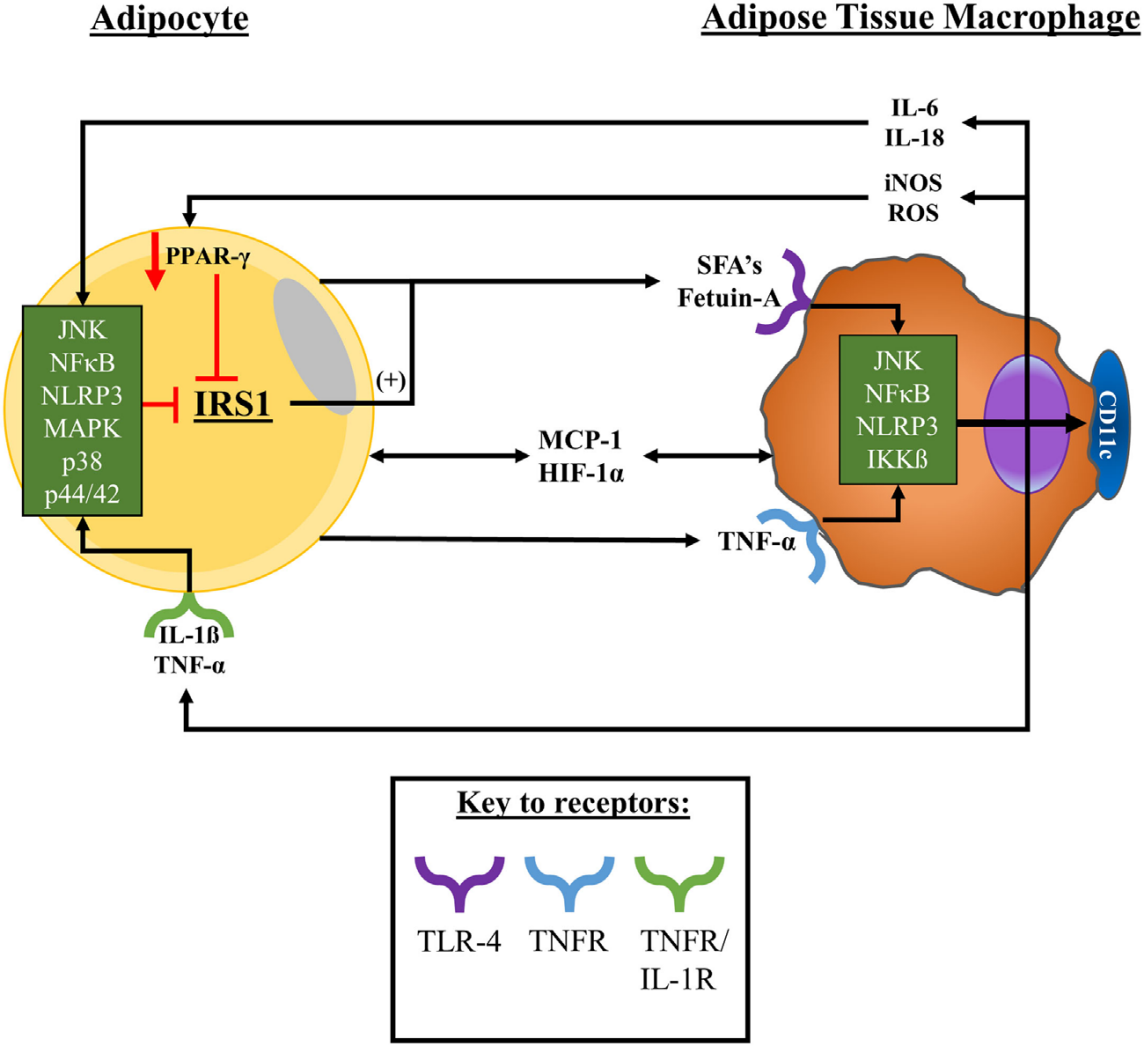
Adipocyte–macrophage interactions with obesity. With obesity, adipocytes expand promoting a state of proinflammation, ER stress, and tissue hypoxia, promoting adipocyte apoptosis/necrosis. Upon these changes in obese adipose tissue macrophages are signalled into the tissue by hypoxic, chemokine, and cytokine signals to surround dysfunctional adipocytes. Macrophages in obese adipose tissue release, and are exposed to, TNF-α and MCP-1—among other factors, including SFAs—which collectively promote the activation of a variety of stress signalling cascades. Consequently, adipose-resident macrophages adopt a pro-inflammatory secretory profile and upregulate their surface expression of CD11c. As a result, adipocytes become exposed to secreted factors such as TNF-α and MCP-1, exacerbating the pro-inflammatory microenvironment of the tissue and potentiating the activation of stress signal cascades within adipocytes. These interactions promote a feed-forward recruitment of monocytes into the tissue and their pro-inflammatory polarisation as well as impairing tissue insulin sensitivity that potentially promotes the release of FFAs into the microenvironment. Abbreviations: CD, cluster of differentiation; ER, endoplasmic reticulum; FFAs, free-fatty acids; HIF-1α, hypoxia-inducible factor-1α; IKKβ, inhibitor of nuclear factor kappa B kinase subunit beta; IL, interleukin; IL-1R, interleukin-1 receptor; iNOS, inducible nitric oxide synthase; IRS-1, insulin receptor substrate 1; JNK, c-Jun *N*-terminal kinases; MAPK, mitogen-activated protein kinase; MCP-1, monocyte chemoattractant protein 1; NFκB, nuclear factor kappa B; NLRP-3, NLR family pyrin domain containing 3; PPAR-γ, peroxisome proliferator-activated receptor gamma; ROS, reactive oxygen species; SFAs, saturated fatty acids; TNFR, tumour necrosis factor receptor; TNF-α, tumour necrosis factor-α.

In summary, a number of factors including adipose tissue cellular composition and secretions plus broader anatomical and physiological characteristics of the tissue have been found to modulate metabolic and inflammatory processes and influence macrophage phenotype and function. However, due to adipose sampling in humans being a “snap-shot” of the dynamic changes that the tissue undergoes with obesity, further work is required to fully understand these interactions. A summary of the likely interactions between adipocytes and adipose-resident macrophages with obesity is presented in Figure 2.

## A Broader Perspective on Adipose Tissue Immunology

Adipose tissue dysfunction is the result of a complex interaction between all cell types within the tissue. Understanding this interplay has been the subject of intensive investigation, which will first be discussed in the context of obesity as a foundation for understanding the function of adipose tissue with ageing. The following sections will summarise research examining different immune cell populations in adipose tissue in the context of obesity, considering cells of the weight of evidence (most to least) supporting their role within dysfunctional adipose tissue.

### Neutrophils in Obesity-Associated Adipose Tissue Dysfunction

In mice fed a high-fat diet, the accumulation of neutrophils (identified in mice as Ly6g+CD11b+) occurs in visceral, but not subcutaneous, adipose tissue after 3 days (89). Neutrophils are 20-fold more abundant in adipose tissue from mice fed a high-fat diet compared to chow-fed mice, accounting for ~2% of total stromal cells (89–91). The accumulation of neutrophils in adipose tissue in obese mice leads to elevated elastase production (89). *In vitro* experiments have shown that increased exposure to elastase can contribute to IRS-1 downregulation, resulting in impaired glucose tolerance and insulin resistance (92). Further, systemic and intraperitoneal glucose tolerance has been shown to be impaired in mice injected with elastase compared to those treated with an elastase inhibitor. Neutrophil-derived elastase also imparts pro-inflammatory actions on murine intraperitoneal macrophages *via* TLR-4 signalling, upregulating *TNF-α, IL-1β*, and *IL-6* gene expression, potentially resulting in a feed-forward recruitment of neutrophils and M1-like macrophages into adipose tissue (89).

In humans, immunohistochemical analyses (CD66b+ staining) have shown neutrophils to accumulate in the microvasculature of adipose tissue with increasing adiposity, correlating with elevated NFκB and cyclooxygenase-2 (COX-2) staining (93). Further, it has also been suggested that elevated chemokine (C-X-C motif) ligand (CXCL)-2 release from obese adipose tissue may promote neutrophil infiltration (94). However, in humans at least, several questions remain unanswered. For example, whether neutrophil accumulation is an early step in adipose tissue dysfunction and to what extent neutrophils modulate ongoing inflammatory responses with adipose tissue.

### NK-Cells in Obesity-Associated Adipose Tissue Dysfunction

Comprehensive investigations in mice have suggested that a population of NK-cells, uniquely expressing the IL-6 receptor alpha and colony-stimulating factor 1 receptor (IL6Ra+Csf1r+), induced *via* IL-6/STAT-3 (signal transducer and activator of transcription-3) signalling, accumulate with obesity within visceral adipose tissue (95). Ablation of IL6Ra+Csf1r+ NK-cells improves insulin and glucose control and reduces overall adiposity, adipocyte size, macrophage infiltration, and macrophage crown-like structures (95). It has been suggested that IL-15 and aberrant leptin secretion with obesity might instigate an adipose-specific NK-cell expansion and activation in response to high-fat diet overfeeding, stimulating NK-cell IFN-γ, TNF-α, and MCP-1 production, negatively impacting whole-body insulin sensitivity (96–98).

In humans with obesity and type-II diabetes, activated NK-cells accumulate in visceral and subcutaneous abdominal adipose tissue, producing IFN-γ and TNF-α (99–101). Moreover, the increase in IL6Ra+ NK-cells in peripheral blood of people with obesity suggests that obese dysfunctional adipose tissue may cause a pro-inflammatory NK-cell population expansion that spills out into the circulation (95). It has been proposed that adipose-resident macrophages produce CCL-3, CCL-4, and CXCL-10, stimulating the recruitment of NK-cells into adipose tissue, whereupon macrophage-derived IL-15 promotes NK-cell proliferation and activation (102). Once within adipose tissue, NK-cell modulation of MCP-1 expression could potentiate macrophage recruitment and proliferation (60), and finally, through an upregulation of NK-cell-derived TNF-α and IFN-γ, NK-cells may guide M1-like macrophage polarisation. Therefore, NK-cells may be pivotal in the generation of obesity-associated immunometabolic adipose tissue dysfunction.

### B-Cells in Obesity-Associated Adipose Tissue Dysfunction

In lean murine visceral adipose tissue, B-cells constitute 10% of the stromal fraction, increasing to 20% in response to high-fat overfeeding, and this accumulation has been reported to occur before macrophage infiltration (103). B-cell localisation around macrophage clusters may be central to their functional relevance within adipose tissue in obesity, suggesting a role in steering macrophage functionality, perhaps *via* LTB4/leukotriene-B4 receptor 1 (LTB4R1) signalling. LTB4/LTB4R1 signalling promotes leukocyte infiltration into tissues, influences cytokine production, and is increased in obese murine visceral adipose tissue B-cells (103). In support, B-cells promote visceral adipose tissue macrophage recruitment and TNF-α production, *in vivo* and *in vitro*, in mice fed a high-fat diet (104). Moreover, B-cells have also been linked with the accumulation and differentiation of IFN-γ-producing CD4+ and CD8+ T-cells within murine visceral adipose tissue (103, 105). Indeed, CD8+ T-cells have been shown to produce 30% less IFN-γ in B-cell-deficient mice fed a high-fat diet suggesting a role for B-cells in T-cell activation (104, 105). B-cells may also promote the expansion of a senescent population of CD4+CD153+PD-1+ T-cells in murine visceral adipose tissue that, in turn, secrete osteopontin that promotes B-cell IgG production and suppresses IL-10 secretion (105, 106).

In obese human subcutaneous adipose tissue, B-cells comprise <4% of all stromal cells, but localise around macrophage crown-like structures and in the perivascular space (107). In obese human adipose tissue, a ~3-fold increase in the expression of the B-cell marker *B220* mRNA has been reported (108), although flow cytometric analysis of human subcutaneous adipose tissue has shown that very few B-cells are present in adipose tissue (109). Contradictory findings could be an artefact of methodology, indicating that further work in humans, employing a combination of methodological approaches, is warranted. In summary, B-cells may play a role in the early stages of obesity-induced adipose tissue dysfunction, modulating the functions of other cells within the stromal fraction, promoting a pro-inflammatory microenvironment.

### T-Cells in Obesity-Associated Adipose Tissue Dysfunction

Within lean human adipose tissue, T-cells represent ~10% of the stromal fraction (one-third CD8+ T-cells and two-thirds CD4+ T-cells). More than half of the CD4+ T-cell compartment within murine adipose tissue are regulatory T-cells (110). Studies in humans have shown that T-cells are recruited into adipose tissue in response to adipocyte-derived CCL-20 binding to CCL-6 on the surface of T-cells, as well as CCR-5 to RANTES interactions (109, 111, 112). Moreover, adipose-resident T-cells display a visceral adipose tissue-specific antigen-driven expansion, suggesting a signal within the tissue promotes their clonal expansion. A potential signal has been suggested to derive from B-cells, which may infiltrate or respond to dysfunctional adipose tissue earlier than T-cells (105, 113, 114). T-cells typically exhibit a differentiated or activated phenotype shown by low-surface expression of CD62L and high expression of CD25 in murine and human adipose tissue, respectively (35, 113).

#### CD8+ Cytotoxic T-Cells in Obesity-Associated Adipose Tissue Dysfunction

In mice fed a high-fat diet, CD8+ T-cells are the predominant T-cell subpopulation to accumulate in adipose tissue. The early selective recruitment of CD8+ cells into visceral adipose tissue in mice occurs in parallel with a reduction in blood CD8+ T-cells in the first 2 weeks of overfeeding (113). Mouse models also show that adipose-resident CD8+ T-cells have an activated CD62L−CD44+ effector phenotype, which is brought about, *in situ*, by MCP-1 release from adipocytes (113, 115). Co-culturing CD8+ T-cells with obese murine adipocytes has been shown to initiate, potentiate, and maintain the inflammatory response by instigating the infiltration of macrophages towards adipocytes, followed by their activation by CD8+ T-cell-derived MCP-1 (113). Moreover, selective depletion of CD8+ T-cells in obese mice has been shown to improve metabolic health and reduce local tissue and serum levels of IL-6 and TNF-α (113).

In obese humans, CD8A mRNA expression is increased in visceral adipose tissue (113, 116), whilst in subcutaneous adipose tissue CD8+ T-cells adopt an activated phenotype compared to blood (35, 109). As serum MCP-1 is substantially elevated in patients with type-II diabetes and is a potent activator of CD8+ T-cells, MCP-1 may be a cause of elevated adipose tissue CD8+ T-cell activation with obesity or metabolic disease (115, 117). The stimulus for CD8+ T-cell migration into obese adipose tissue is yet to be confirmed *in vivo*, in humans, although significantly elevated CCL-20 release by obese adipose tissue has been observed *in vitro* (109). In the circulation, CD8+ T-cells from obese individuals express elevated levels of TNF-α, IFN-γ, and RANTES. Importantly, IFN-γ has been found to inhibit insulin-mediated upregulation of fatty acid synthase (FAS) and lipoprotein lipase and was associated with a downregulation of phosphatidylinositol 3-kinase regulatory subunit alpha expression (a key component of insulin signalling) (109). These observations suggest a modulatory capacity for CD8+ T-cells in adipose tissue metabolic health by influencing insulin-mediated triglyceride storage (109).

#### CD4+ Helper T-Cells (Th Cells) in Obesity-Associated Adipose Tissue Dysfunction

CD4+ T-cells accumulate within human subcutaneous adipose tissue with obesity, exhibiting an activated CD25+ phenotype (35). Recent findings suggest that circulating CD4+ T-cells adopt an effector memory (CXCR-3+CD62L−) phenotype with human obesity in response to a metabolically driven adaptation within T-cells *via* the p110δ subunit of phosphoinositide 3-kinase (PI3K) (118). Indeed, lipid activation of this subunit is also associated with sustained activation of T-cells, indicating that dendritic cell-independent, metabolically driven activation could drive chronic activation within adipose tissue (119, 120). The Th cell balance has the potential to play an important role in adipose tissue inflammatory disorders. For example, in mice, IFN-γ secretion is increased mainly as a consequence of Th cell (Th-1) cell predominance within visceral adipose tissue upon high-fat diet overfeeding (112, 114). The direct effect of IFN-γ in obesity induced by a high-fat diet includes impaired insulin signalling and the promotion of macrophage infiltration and crown-like formations *via* MCP-1 (121). In addition, IFN-γ from Th-1 cells instigates CXCL-10 release from 3T3-L1 adipocytes, providing a positive feedback recruitment loop (122). Moreover, IFN-γ secretion, when combined with TNF-α, can lead to chronic activation of NFκB signalling (123). However, Th-2 cells could serve to promote M2-like macrophage maturation through their production of IL-4 and IL-13 (124). Human and murine research indicates that CD4+ T-cells within both visceral and subcutaneous adipose tissue not only control their own recruitment, activation, and differentiation but are also influenced by other tissue-resident immune cells (i.e., B-cells) and adipocytes. Consequently, CD4+ T-cells have both direct and indirect roles in obesity-associated adipose tissue immunometabolic dysfunction.

#### Regulatory T-Cells in Obesity-Associated Adipose Tissue Dysfunction

Regulatory T-cells represent more than half of all CD4+ T-cells in lean murine adipose tissue, and in obesity, regulatory T-cells accumulate around macrophages, produce IL-4 and IL-10, and promote Th-2 T-cell polarisation (110). Regulatory T-cells also suppress adipocyte-derived inflammatory markers and restore metabolic health by promoting translocation of glucose transporter-4 to the cell membrane, countering the effects of TNF-α. However, adipose-resident regulatory T-cells appear to decline with increasing duration of obesity in mice (125). It is thought that the decline in regulatory T-cell numbers is a result of IFN-γ-induced impairments in proliferative capacity in visceral adipose (125). These changes occur in parallel with insulin resistance in mice and humans (110, 113, 114). In human obesity—which occurs over a much longer time-span than in mice—it has been shown that there is a substantial increase in regulatory T-cells within subcutaneous adipose tissue, and this could be a compensatory mechanism to counter adipose tissue inflammation (35). However, a proportional decline in regulatory T-cells has also been reported within the adipose tissue stromal fraction in obesity (126). These observations suggest that regulatory T-cells may limit ongoing pro-inflammatory events that occur with obesity-induced adipose tissue dysfunction. However, it is possible that with chronic local inflammation, regulatory T-cell proliferation is impaired and numbers decline.

#### iNKT Cells in Obesity-Associated Adipose Tissue Dysfunction

Within lean human and murine adipose tissue, iNKT cells typically express low levels of CD4 and NK1.1 and secrete large amounts of IL-4 and IL-10, demonstrating an anti-inflammatory capacity (127). Indeed, with obesity, iNKT cells may initially provide an anti-inflammatory function to limit the ongoing pro-inflammatory response driven by other infiltrating leukocytes. However, with obesity, iNKT cells decline substantially although the reason for this decline is unclear (127). Further, in mice, iNKT cell accumulation in adipose tissue is limited with adipocyte CD1d knockout and, in turn, these animals exhibit a poor metabolic profile (128). However, it has been suggested that CD1d knockout models may reduce IFN-γ production, independent of iNKT cells (128). In addition, recent investigations have shown that iNKT cells have a role in adipocyte browning and modulating regulatory T-cell responses in mice, promoting the anti-inflammatory actions of regulatory T-cells (129, 130). In non-obese humans, visceral adipose tissue is also enriched with iNKT cells, and these cells exhibit a distinct Th-2 cytokine profile (131, 132). However, with obesity, the proportion of iNKT cells within the adipose tissue stromal fraction declines substantially, in tandem with the development of metabolic syndrome and inflammation, as observed in obese mice (128, 131, 132). In summary, iNKT cells likely assist in the regulation of a healthy adipose tissue microenvironment in a similar way to regulatory T-cells and eosinophils (see Eosinophils in Obesity-Associated Adipose Tissue Dysfunction). Therefore, a reduction in iNKT cells with obesity might contribute to the diminished anti-inflammatory signals within dysfunctional adipose tissue.

### Dendritic Cells in Obesity-Associated Adipose Tissue Dysfunction

Dendritic cells accumulate in adipose tissue of mice fed a high-fat diet and likely contribute to the pro-inflammatory microenvironment *via* macrophage recruitment and IL-6 production (133, 134). Myeloid dendritic cells may also promote Th cells to adopt a Th-1 phenotype, which contribute to IFN-γ production (133, 135–137). However, in mice, both the flow cytometric analyses and knockout models commonly utilised are subject to criticism due to their non-specificity for “true” dendritic cells (133, 138). It has recently been shown that type-I IFN signalling is pivotal to the development of obesity-associated metabolic disease in mice, and therefore, plasmacytoid dendritic cells have been implicated in diet-induced obesity (139). Indeed, in mice fed a high-fat diet, plasmacytoid dendritic cells have been shown to increase ~3-fold, possibly as a result of elevated recruitment and activation with obesity by the adipokine, chemerin. In turn, dendritic cell accumulation has been suggested to enhance type-I IFN signalling, leading to IFN-β-induced MCP-1 production and macrophage recruitment/proliferation (60, 133, 139–143). In humans, distinct CD1c+ myeloid dendritic cell populations have been identified within subcutaneous, but not visceral adipose tissue, and the number of these cells correlates positively with BMI (138). Moreover, dietary lipids modulate the abundance of dendritic cells within lymphoid structures in adipose tissue, and as dendritic cells communicate with other lymphoid cells *via* lipid-derived molecules, dendritic cells may modulate adipose tissue immunometabolic responses (144, 145).

### Eosinophils in Obesity-Associated Adipose Tissue Dysfunction

Eosinophils in the abdominal adipose tissue of mice have a role in improving insulin sensitivity and stimulating anti-inflammatory responses through the production of IL-4 (146–148). Mice fed a high-fat diet exhibit a decline in eosinophil numbers within adipose tissue (147). In addition, eosinophils have been estimated to produce up to 90% of the IL-4 found within murine adipose tissue, promoting M2-like macrophage polarisation and tyrosine hydroxylase expression. In turn, macrophage tyrosine hydroxylase expression drives the production of catecholamines by macrophages, triggering the expression of uncoupling protein 1 in white adipose tissue (148–150). Moreover, it has been shown that mice fed a high-fat diet and injected with eosinophils intravenously do not accumulate adipose-resident macrophages (147). Further, obese eosinophil knockout mice demonstrate significantly greater degrees of insulin resistance compared to wild-type mice (147).

### Mast Cells in Obesity-Associated Adipose Tissue Dysfunction

Obesity in mice is associated with an accumulation of mast cells in adipose tissue and higher levels of tryptase in serum—a granule released by mast cells (151). In mice fed a high-fat diet, mast cells have been reported to accumulate in visceral adipose tissue and may contribute to the development of obesity through the stimulation of adipocyte protease expression, promoting microvessel growth, enabling leukocyte infiltration, and adipose tissue expansion (151, 152). In humans with obesity and type-II diabetes, pharmacological stabilisation of mast cells to prevent degranulation leads to improvements in a number of parameters, including glycosylated haemoglobin, fasting blood glucose, total cholesterol, low-density lipoprotein, triglycerides, and high-density lipoprotein, independent of changes to BMI (153). Therefore, despite contradictory findings in murine research, study on humans indicates a potential role for mast cell dysregulation in the aetiology of metabolic diseases. However, whether this is a result of adipose-resident mast cells specifically is yet to be established.

### Summary of the Cellular Changes Associated with Obesity-Induced Adipose Tissue Dysfunction

**Figure 3:**
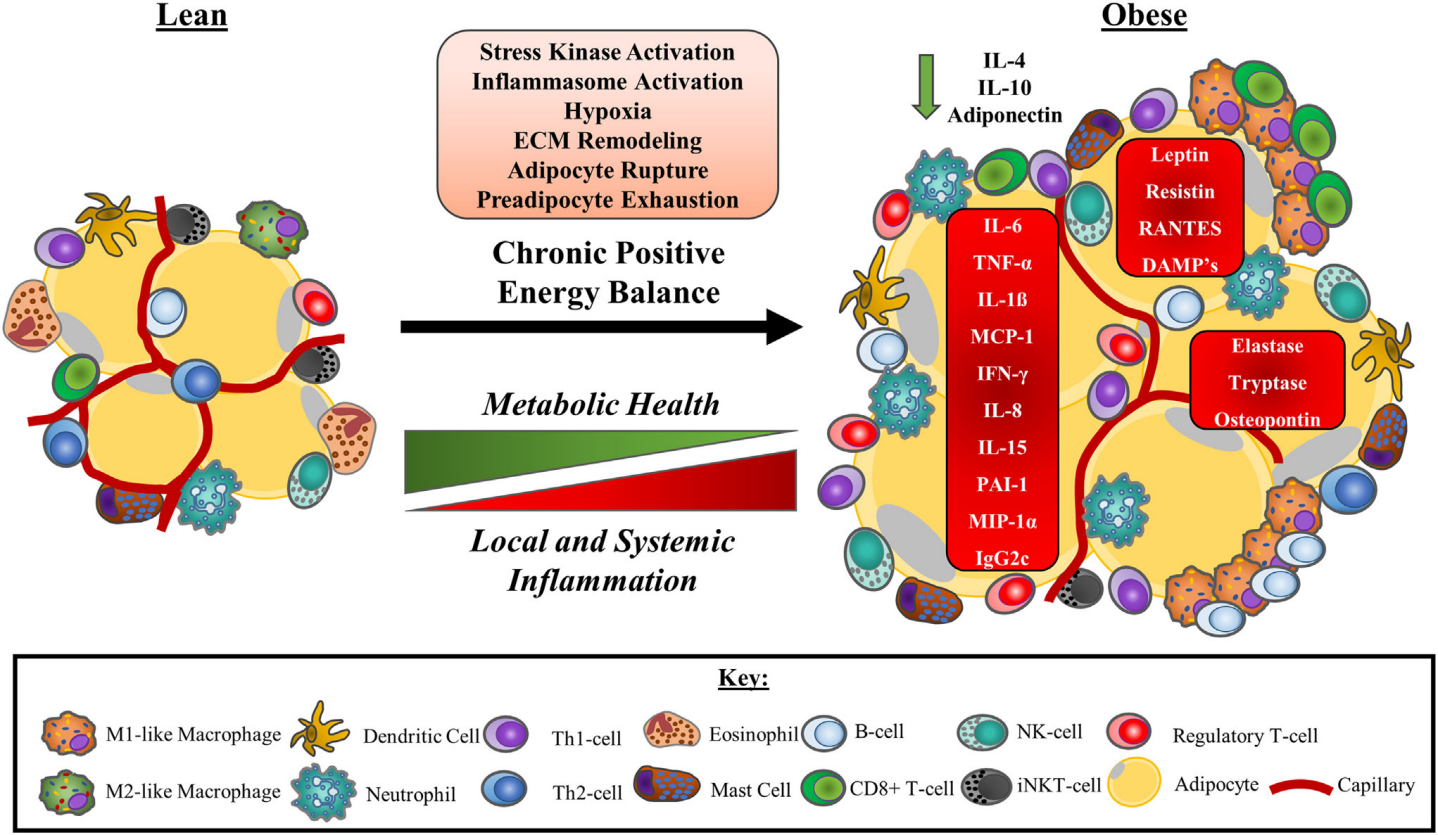
A model of obesity-driven immunological changes within adipose tissue. Under a chronic state of positive energy balance, adipose tissue undergoes a multitude of changes that include the hypertrophic expansion of adipocytes without concurrent angiogenic responses. The consequences of these adaptations include local tissue hypoxia as a result of reduced tissue blood flow and impaired oxygen delivery. The rapid expansion of adipocytes also leads to unstable adipocytes prone to rupturing, releasing their lipid contents. This instability is further exacerbated by an exhaustion of the preadipocytes required to facilitate adipose tissue expansion. The net result of these physiological changes to adipocytes is the release of DAMPs and other signalling molecules that initiate stress kinase activation and promote monocyte infiltration to manage the remodelling of the ECM. Neutrophils are the first cells to accumulate in adipose tissue, followed by a range of other immune cells, most notably, B-cells, CD8+ T-cells, and M1-like/intermediate, or “double-negative” (CD11c−CD206−) macrophages that surround unstable adipocytes in crown-like structures. As a consequence, a Th-1 cell and M1-like macrophage secretion profile predominated and some anti-inflammatory immune cells such as eosinophils and iNKT-cells leave the tissue. Other anti-inflammatory cells, including regulatory T-cells, may increase to compensate. Pro-inflammatory cytokines and adipokines dominate the tissue microenvironment potentiating the activation of stress kinases and inflammasomes. Local inflammation reduces adipose tissue insulin sensitivity, impairing metabolic health and promoting ectopic lipid deposition. Pro-inflammatory adipose tissue contributes to the chronic, low-grade inflammation characteristic of obesity, and metabolic disease. Abbreviations: CD, cluster of differentiation marker; DAMP, damage-associated molecular pattern; ECM, extracellular matrix; IFN-γ, interferon gamma; Ig, immunoglobulin; IL, interleukin; iNKT-cell, invariant natural killer T cell; MCP-1, monocyte chemotactic protein 1 (CCL-2); MIP-1α, macrophage inflammatory protein 1 alpha (CCL-3); PAI-1, plasminogen activator inhibitor 1; RANTES, regulated on activation, normal T-cell expressed and secreted (CCL-5); Th cell, helper T cell; TNF-α, tumour necrosis factor-α.

Adipose tissue dysfunction with obesity is associated with changes to the numbers and/or function of a variety of immune cells. With this ongoing immunological dysregulation, adipose tissue adopts a pro-inflammatory bias, dramatically impacting the metabolic health of the tissue. Consequently, dysfunctional adipose tissue influences whole-body immunometabolic health contributing to the generation of diseases including type-II diabetes and cardiovascular diseases. A summary of obesity-associated changes to adipose tissue-resident immune cell populations is presented in Figure 3.

## Immunological Ageing and Adipose Tissue

Most physiological systems become dysfunctional with ageing due to accumulations of cellular changes that contribute to the onset of disease. Nine hallmarks of ageing have been proposed characterising age-associated whole-body dysfunction, which include genomic instability, telomere attrition, epigenetic alterations, loss of proteostasis, dysregulated nutrient sensing, mitochondrial dysfunction, cellular senescence, stem cell exhaustion, and altered intracellular communication (15). The physiological stresses induced by obesity also cause a range of cellular and whole-body deteriorations that underpin the pathophysiology of adipose tissue accumulation and dysfunction. Many of these obesity-associated changes overlap with the hallmarks of ageing; thus, it has been proposed that obesity could be considered an accelerated model of ageing (13, 28, 154, 155).

Ageing is associated with a decline in immune function, referred to as immunosenescence, which is thought to contribute to chronic, low-grade inflammation or “inflammageing” (16, 156–159). The broader consequences of an ageing immune system are increased susceptibility to infections and cancer in combination with weaker responses to novel antigens, including those administered by vaccination (160). Immunosenescence is best characterised and most marked among cells of the adaptive immune system. However, a common general observation is that lymphoid cell numbers decline (along with their proliferative capacity), whereas myeloid cell numbers typically increase, but only some innate immune cells exhibit age-associated dysfunction (156). The most robust hallmarks of immunosenescence are alterations to the T-cell pool in peripheral blood. These changes are, in-part, not only due to thymic involution limiting the output of naive T-cells but also due to a lifetime of antigen exposure, which drives the differentiation of naive T-cells into memory T-cells (160). Thus, ageing is associated with an accumulation of pro-inflammatory effector memory T-cells and a decline in naive T-cells in peripheral blood (160–162).

Obesity, as with ageing, is associated with increased susceptibility to infections and an inability to mount effective immune responses to novel antigens (163–166). Thus, obesity might promote immunological decline through several mechanisms, with dysfunctional adipose tissue as a potential-driving factor. For example, obesity might exacerbate immunosenescence by promoting activation and differentiation of immune cells passing through the adipose tissue microvasculature, which could have implications for the phenotype and function of cells found in peripheral blood and other tissues. In addition, immune cell accumulation in adipose tissue promotes differentiation into pro-inflammatory cytokine-producing cells, which likely drives inflammageing. Exacerbated inflammation influence processes early in the immune response to novel pathogens, such as antigen recognition, processing, and presentation, but might also impair the ability to control latent or chronic infections and undergo processes such as wound healing. Experimental evidence supports some of these ideas, suggesting that adipose tissue dysfunction in obesity promotes immunological ageing (105).

Although there is less research specifically examining adipose tissue in the context ageing compared to obesity, a role for adipose tissue in late-onset disease development has been established. Ageing is associated with increased abdominal and ectopic adipose tissue accumulation, which is linked with the onset of frailty and cardiometabolic disease (167). Moreover, organelle stress and the accumulation of senescent cells (p16INK4a+) within adipose tissue that secrete IL-6, IL-8, and TNF-α further contribute to a pro-inflammatory, dysfunctional adipose tissue with age (168, 169). However, comparatively little is understood about immune cell populations within aged adipose tissue, especially in humans. Thus, the remaining sections will review literature examining the accumulation of different immune cells in adipose tissue with ageing, beginning with cells where there is most evidence, followed by cells that have been examined least.

### Macrophages in Aged Adipose Tissue

**Figure 4:**
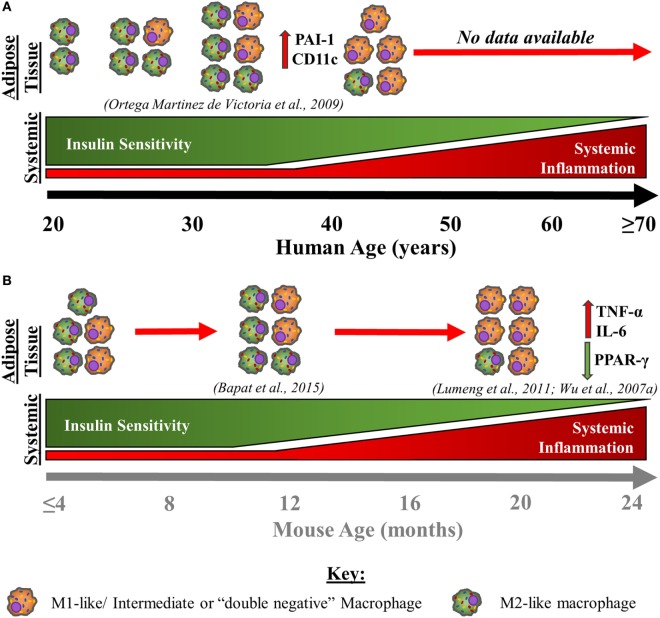
Age-associated changes in adipose tissue macrophages and immunometabolic health in humans **(A)** and mice **(B)**. This schematic is a theoretical representation based on the limited empirical evidence that is available. With advancing age in humans **(A)**, subcutaneous adipose tissue macrophage content increases steadily until 31–33 years whereupon numbers slightly decline, until 45 years old (174). Although absolute adipose tissue macrophage content declines from around the age 31–33 years old until 45 years old, at least, the presence of more pro-inflammatory activated macrophages [M1-like/intermediate, or “double-negative” (CD11c−CD206−) macrophages] increases, which is associated with a decline in insulin sensitivity. With advancing age, insulin sensitivity continues to decline, while low-grade systemic inflammation begins to steadily increase from middle age into old age. With advancing age in mice **(B)**, an increase in pro-inflammatory macrophages within visceral adipose tissue that produce TNF-α and IL-6 and have downregulated PPAR-γ expression is also observed up to 18–24 months, at least (51, 170). Further, in middle-aged mice (44 weeks old), “double-negative” (CD11c−CD206−) and M2-like macrophages are largely unchanged or are modestly elevated, while M1-like macrophages exhibit a slight decline (172). Therefore, it is theorised that there is a continued pro-inflammatory macrophage activation/polarisation with advancing age in humans, independent of changes in absolute counts within the adipose tissue. Further, the same pattern of events appears to occur within murine adipose tissue, although the age at which this shift to a pro-inflammatory bias occurs is unknown. The similarities between data from aged mice and humans indicate that macrophages adopt a predominantly pro-inflammatory M1-like/intermediate, or “double-negative” (CD11c−CD206−) phenotype into old age, contributing to the increased systemic inflammation and impaired metabolic health exhibited by older individuals. Abbreviations: CD, cluster of differentiation marker; IL, interleukin; PAI-1, plasminogen activator inhibitor 1; PPAR-γ, peroxisome proliferator-activated receptor gamma; TNF-α, tumour necrosis factor-α.

Very few studies have investigated the effects of ageing on adipose tissue macrophage populations to date; however, the available data support a role for macrophages in age-associated adipose tissue dysfunction. This concept is discussed below and summarised in Figure 4. In visceral adipose tissue from old non-obese mice, it has been shown that the absolute numbers of adipose-resident macrophages do not increase substantially but may decline in proportions given the expanded stromal vascular fraction with age. However, with age, visceral adipose tissue macrophages have a predominantly pro-inflammatory, so-called “double-negative” (CD11c−CD206−) phenotype producing IL-6 and TNF-α and down regulating *PPAR-γ* (51, 170). Furthermore, peritoneal macrophages cultured with adipose-derived conditioned media induces M1-like polarisation, indicating that the products of adipose tissue have a direct impact on immune cells (170). Age-associated adipocyte-derived inflammation likely comes about by the activation of the NFκB signalling pathway (51). As a result, NFκB activation may act as a stimulus for the pro-inflammatory polarisation of adipose tissue macrophages, supporting the idea that changes within adipose tissue, independent from total adipose tissue mass, drive age-associated adipose tissue inflammation (171). These observations in mice likely correspond to those made in obese adipose tissue from humans (Figure 1) but experimental evidence is limited at present. Moreover, another study in middle-aged (11 months old) mice suggests that the proportions of “double-negative” (CD11c−CD206−) and M2-like macrophages are largely unaltered, whereas M1-like macrophages decline slightly when compared to young murine visceral adipose tissue (172). Note that these 11-month-old mice are representative of humans aged 30–40 years (173).

Sympathetic neuron-associated, catecholamine-degrading macrophages which impair lipolysis have also been reported to accumulate in aged murine visceral adipose tissue (175). With obesity, these sympathetic neuron-associated macrophages appear to infiltrate the tissue selectively (87). However, with ageing, these cells appear to accumulate in adipose tissue *via* proliferative mechanisms, *in situ* (175). The metabolic effects of these cells are due to NLRP-3 activation, which upregulates growth differentiation factor 3 (GDF3) expression within macrophages (175). GDF3 interacts with adipocytes to inhibit lipolysis and stimulates the expression of enzymes that oxidise and remove catecholamines (87, 175). Therefore, in the context of ageing, sympathetic neuron-associated macrophages likely regulate whole-body metabolism, inhibiting lipolytic signalling and free-fatty acid mobilisation as with obesity, contributing to immunometabolic dysfunction both locally and systemically.

In humans, only one study has investigated how ageing influences adipose tissue macrophages. Metabolically healthy Pima Indians, aged 18–44 years, were assessed for insulin sensitivity and subcutaneous adipose tissue macrophage content. Adipose tissue macrophage number increased until ~31–33 years old, plateauing or slightly declining thereafter, but with ageing, adopted an activated phenotype as shown by plasminogen activator inhibitor 1 and *CD11c* mRNA expression (Figure 4). The expression of these two macrophage activation markers was also associated with a decline in insulin sensitivity, independent of adiposity. Further, a negative relationship was observed between advancing age and whole-body insulin sensitivity (*r* = −0.23, *P* = 0.07). These findings suggest that although adipose-resident macrophages may decline or plateau in middle age, activation of these cells might impart an insulin-desensitising effect (174). Although this study offers the only direct assessment of adipose-resident macrophage populations with advancing age in humans, there are several factors that should be considered. For example, this study provides data up until middle age only and was conducted in a specific population with unique metabolic, genetic, and lifestyle characteristics. In addition, examining adipose-resident macrophages using gene expression and immunohistochemical analysis has some limitations (see Table 1).

The stimulus for age-associated macrophage pro-inflammatory polarisation is unclear, but endoplasmic reticulum stress, as shown in obese adipose tissue and adipose tissue macrophages, has been implicated. For example, it has been shown in aged murine adipose tissue stromal cells that endoplasmic reticulum stress promotes an inflammatory environment, elevating IL-6, MCP-1, and TNF-α production within adipose tissue, potentially in response to altered autophagy (176, 177). Ageing is associated with decreased vascularisation as a result of impaired angiogenesis, supporting local hypoxia, which might drive adipose tissue macrophage accumulation (36, 178–180). Ageing is also associated with reductions in the mitochondrial cytochrome *c* oxidase subunit 5B (COX5B) component of complex IV within adipocytes, which represses HIF-1α (181). Therefore, reductions in COX5B in human visceral adipose tissue with age both elevates HIF-1α and intracellular lipid storage as a result of decreased fatty acid oxidation, promoting adipocyte enlargement, *in vitro* (181). If this hypertrophic expansion was to continue, stress signals prompting macrophage infiltration could be released, as is observed in obesity (53, 79, 182).

### Regulatory T-Cells in Aged Adipose Tissue

As with obesity, regulatory T-cells increase in the adipose tissue of old compared to young mice (110, 170, 172). The expanded population of these regulatory T-cells is transcriptionally more closely related to adipose-resident conventional T-cells than splenic regulatory T-cells (i.e., selective enrichment for *PPAR-γ* gene expression, among others), indicating that their characteristics may be driven, in part, by their location within the adipose tissue (172). However, unlike in obesity, an increase in regulatory T-cells in aged murine adipose tissue is linked with a decline in metabolic function independent of macrophage accumulation (172). These observations indicate that, in aged adipose tissue, a reduction in regulatory T-cells may be protective, because the immune profile of adipose tissue regulatory T-cell knockout mice is shifted towards those of young mice (172). Moreover, *ex vivo* adipose tissue basal glucose uptake was nearly twice as high for adipose-resident regulatory T-cell knockout mice compared to control tissues, supporting a causal association between adipose tissue regulatory T-cells and age-associated insulin resistance (172).

Although it is unclear whether the function of regulatory T-cells with ageing in humans is impaired, studies in aged mice indicate that these cells retain their suppressive capacity (172, 183). As a degree of inflammation is essential for the maintenance of healthy adipose tissue, the substantial increase in adipose tissue regulatory T-cells combined with the retention of their suppressive capacity may be an indication of overcompensation, impacting the immunometabolic properties of aged murine adipose tissue (26, 172). Although the observations concerning regulatory T-cells within aged murine adipose tissue provide evidence that age- and obesity-associated insulin insensitivity may be orchestrated by unique immune cell populations, further work in the adipose tissue of older humans is required to better understand this immunometabolic interplay. Additional work in older mice is also warranted as the animals examined by Bapat et al. (172) were substantially younger than those examined in other studies investigating age-associated adipose tissue immunological dysfunction (51, 170, 172) (see Figure 4). Nonetheless, recent evidence offers some support for a non-obesity-dependent regulatory T-cell-driven, late-onset metabolic deterioration, called type-IV diabetes (172).

### B-Cells in Aged Adipose Tissue

It has been shown that the visceral adipose tissue of obese, old mice is enriched with a specific B-cell population compared to young, obese mice, and these cells differ from splenic B-cells (184). Co-culturing splenic B-cells with visceral adipocytes from old, obese mice caused B-cells to adopt an age-associated phenotype (184). These B-cells, which had been altered by adipose tissue, had higher levels of basal inflammation and NFκB protein within the nuclear extract, as has been shown with peripheral blood B-cells from older humans (185). In addition, the visceral adipose tissue of old mice has also been shown to be enriched with IgG2c (a mouse-specific IgG isoform), an observation that has also been made in obese mice (104, 184). It was proposed by Frasca and Blomberg (184) that increased hypoxia and *CCL-2* expression, along with increased adipocyte-derived TNF-α, IFN-γ, IL-6, and IL-21, prompted an obesity-driven accumulation of pro-inflammatory B-cells, which is exacerbated with ageing (184). Nonetheless, it should be noted that older mice in this study were heavier than younger mice and had more visceral adiposity. Thus, it is difficult to tease apart the effects of ageing or obesity in this work. Other evidence suggests that metabolic secretions released from dysfunctional adipose tissue with age, rather than typical pro-inflammatory factors, contribute to B-cell dysfunction. For example, it has been shown that incubation of B-cells from young mice with leptin induces STAT-3 signalling within B-cells, leading to TNF-α and IL-6 production, possibly contributing to the elevated production of these cytokines in aged murine adipose tissue (51, 171, 186).

### CD4+ Th Cells and CD8+ Cytotoxic T-Cells in Aged Adipose Tissue

CD4+ T-cells within the visceral adipose tissue of obese mice display an “aged” phenotype and accumulate 4-fold with obesity (105, 170). High-fat overfeeding in mice causes an accumulation of CD4+ T-cells in the visceral adipose tissue expressing markers associated with senescence such as CD153+PD-1+CD44^high^CD4+ T-cells in visceral adipose tissue. These cells secrete osteopontin, causing local inflammation (105). Moreover, CD4+ T-cells expressing markers such as CD153 also accumulate in the circulation with age in mice, perhaps linking changes in blood to changes in adipose tissue, or *vice versa* (187). Compared to the 4-fold increase in CD4+ T-cells in aged murine visceral adipose tissue, CD8+ T-cells exhibit a 7-fold increase, in parallel with an increase in *CCR-5* gene expression, which is involved in the migration and homing of effector T-cells (170, 188). However, whether adipose tissue CD8+ cells impart a pro-inflammatory function with age is unclear. CD8+ T-cells within aged adipose tissue have not been studied in isolation; although when adipose tissue stromal cells from aged mice were cultured *ex vivo*, no difference was found in the production of IFN-γ compared to young mice (170). Although there was a greater production of TNF-α from aged murine adipose tissue, this was attributed to macrophages (170). It is, therefore, currently unclear what role CD8+ T-cells play in aged adipose tissue. Furthermore, it is unclear what signal promotes this CD8+ T-cell expansion within aged adipose tissue and whether this contributes to, or merely reflects, the increased CD8:CD4 T-cell ratio in the circulation of older individuals (189).

### Under-investigated Cells in Aged Adipose Tissue

Neutrophils, eosinophils, mast cells, dendritic cells, NK-cells, and iNKT cells have a role in obesity-associated adipose tissue dysfunction. Indeed, obesity is associated with alterations in the numbers, proportions and functions of many cell types, either resulting in a pro-inflammatory change (i.e., neutrophils, mast cells, dendritic cells, NK-cells) or an anti-inflammatory change (i.e., eosinophils and iNKT cells). These alterations contribute towards, or occur in response to, the immunometabolic dysfunction within adipose tissue caused by obesity. However, currently, there is no direct evidence for a similar role for these cells within adipose tissue in an ageing context.

Although no research has specifically examined neutrophils in aged adipose tissue in mice or humans, elevated *COX-2* mRNA has been shown in visceral adipose tissue from older mice, which may indicate neutrophil activation in response to elevated basal prostaglandin E2 (PGE_2_) expression (51, 93, 190, 191). Moreover, neutrophils migrate through tissues secreting proteases (e.g., elastase) in response to homing signals including, among others, leptin, and aberrant secretion of this adipokine with age may promote neutrophil entry into adipose tissue (192–195). Neutrophils also demonstrate misdirected chemotaxis, with ageing, which, if neutrophils were signalled into adipose tissue in response to aberrant leptin secretions, would potentially exacerbate damage to the tissue, as membrane-bound proteases would be released over a wider area (196). In obese adipose tissue, increased elastase release by tissue-resident neutrophils impacts upon the metabolic health of adipose tissue by interacting with IRS-1 and TLR-4 signalling, impairing insulin sensitivity (89, 92, 197, 198). Therefore, using obesity as a model, neutrophil degranulation as a result of impaired navigation through the adipose tissue may contribute to age-associated adipose tissue dysfunction.

Mast cell and eosinophil numbers and function in adipose tissue has not been investigated in an ageing context. With eosinophils, considering that the number of these cells do not change with ageing in the circulation, it might be speculated that the same is true in adipose tissue (199). However, in obesity, the decline in adipose tissue eosinophils in mice might promote a pro-inflammatory macrophage predominance due to less eosinophil-derived IL-4 and IL-10. Subsequently, a loss of eosinophil-derived anti-inflammatory signals could contribute to fewer M2-like macrophages and the increase in M1-like or “double-negative” (CD11c−CD206−) macrophages with ageing in visceral adipose tissue. If mast cells are shown to be present in aged adipose tissue, the reported increase in PGE_2_ within aged murine adipose tissue may promote mast cell degranulation, considering that PGE_2_ inhibits the release of mediators that limit mast cell activation (51, 200).

Although no research has examined dendritic cells in adipose tissue with ageing, some evidence suggests that the pro-inflammatory profile of aged murine adipose tissue, as shown by high gene expression for IL-6, TNF-α, and PGE_2_, could influence dendritic cell function (51). PGE_2_ augments IL-23 production in aged murine and human dendritic cells, promoting Th-17 responses, while a pro-inflammatory aged adipose tissue microenvironment could promote NFκB activation within dendritic cells, as is seen in obese adipose tissue (138, 201–205). Dendritic cells from the circulation of older individuals may also have the potential to guide pro-on within adipose tissue due to increased intracellular IL-6 and TNF-α and increased expression of co-stimulatory and activation markers, as is observed in the context of obesity (133, 137, 158, 206). Nonetheless, these observations have yet to be investigated within aged adipose tissue and can, therefore, only represent speculative mechanisms at present.

We are unaware of any research that has investigated the role of NK-cells in aged adipose tissue. However, in mice, aberrant leptin secretions have been suggested to drive specifically activated CD56^dim^ NK-cell polarisation, contributing to elevated TNF-α production and metabolic impairments (95, 96, 194). As well as leptin, the elevated TNF-α within aged murine adipose tissue may also serve to recruit NK-cells as TNF-α is essential for NK-cell recruitment into other organs such as the liver (207). It remains unclear whether the aberrant secretory profile of aged adipose tissue influences NK-cell function. However, as circulating CD56^dim^ NK-cells accumulate in the blood of older people, if these cells were recruited into adipose tissue by TNF-α, leptin, or another yet to be established signal, they may contribute towards pro-inflammatory macrophage polarisation (102, 208–212).

Adipose tissue iNKT-cells have not been investigated with ageing, but considering that IL-4- and IL-10-producing NK1.1+iNKT-cells decline with obesity (127), it is possible that ageing has similar effects. However, given endogenous lipid antigens derived from adipocytes influence the secretion profile of iNKT-cells (127, 213, 214), then interactions between adipocytes and iNKT-cells might contribute towards iNKT-cell dysregulation in aged adipose tissue. For example, loading glycolipids into the CD1d receptor on adipocytes is mediated by intracellular ceramides (127), and sphingolipid ceramide content is higher in adipose tissue from old mice compared to young (51). Thus, in principle, enhanced ceramide-mediated glycolipid loading within aged adipocytes might drive overactivation of iNKT-cells with ageing.

## Summary, Considerations, and Future Directions

Poor metabolic control, insulin resistance, inflammation, and impaired immune function are hallmarks of ageing and also characteristics of obesity. In addition, as outlined in this review, individuals who are obese, or elderly, exhibit similar immunological profiles in adipose tissue (Tables 3–6). Given the overlap between obesity and ageing, it is likely that changes to the numbers and function of some immune cells within obese and aged adipose—especially macrophages—contribute to dysfunction of this tissue. Moreover, it is possible that alterations to the immune profile of obese adipose tissue accelerate local and whole-body ageing. Finally, changes to the numbers and function of some immune cells within obese, and likely aged adipose tissue, whether a cause or consequence of adipose tissue dysfunction, may also underpin the development of metabolic diseases, as shown with obesity. However, there is limited work in an ageing context.

**Table 3:** A comparison of the change in abundance for select immune cell subsets within adipose tissue with age and obesity compared to young and lean, respectively.

	Immune cells within adipose tissue			
	Ageing	Ageing references	Obesity	Obesity references
CD8+ T-cells	↑	(170)	↑	(113)
CD4+ T-cells	↑ ← →	(170)	↑	(35, 105)
Regulatory T-cells	↑(?)	(172)	↑/↓	(35, 125)
B-cells	↑(?)	(184)	↑	(104, 107)
Macrophages				
M1-like/intermediate/DN	↑	(51, 170)	↑	(63, 87)
M2-like	↓	(174, 175)	↓	(35, 65)
Eosinophils	?		↓	(147)
Mast cells	?		↑(?)	(151, 152, 215)
NK cells	?		↑	(95, 97)
iNKT cells	?		↓	(128, 132)
Neutrophils	?		↑	(89, 93)
Dendritic cells	?		↑	(133, 139)

*↓, decrease; ↑, increase; ← →, little or no change or equivocal data; ?, unclear or no evidence; CD, cluster of differentiation marker; DN, double-negative macrophage (CD11c−CD206−); iNKT cell, invariant natural killer T cell; NK-cell, natural killer cell*.

**Table 4:** Changes to adipose tissue and adipocytes with age and obesity compared to young and lean, respectively.

	Broader Tissue-Specific Changes			
	Ageing	Ageing references	Obesity	Obesity references
Adipocyte size	↑	(216)	↑	(31)
Endoplasmic reticulum stress	↑	(176, 177)	↑	(82, 85)
Mitochondrial dysfunction	↑	(181)	↑	(217)
Stem cell exhaustion	↑	(13)	↑	(13)
Tissue blood flow	↓	(180)	↓	(36, 178)
Tissue hypoxia	↑		↑	

*↓, decrease; ↑, increase*.

**Table 5:** Changes in the soluble factors produced and/or released by adipose tissue (adipocytes and cells of the stromal fraction) with age and obesity compared to young and lean, respectively.

	Soluble and secreted factors within adipose tissue			
	Ageing	Ageing references	Obesity	Obesity references
IL-6	↑	(171)	↑	(218)
MCP-1	↑	(170)	↑	(60)
TNF-α	↑	(51)	↑	(27)
IFN- γ	← →	(170)	↑	(113)
IL-10	← →	(170)	↓	(219)
IL-1β	↑	(51)	↑	(79)
Adiponectin	↓← →	(220)	↓	(221)
Leptin	↑	(192, 193)	↑	(222)

*↓, decrease; ↑, increase; ← →, little or no change or equivocal data; IFN-γ, interferon gamma; IL, interleukin; MCP-1, monocyte chemotactic protein 1; TNF-α, tumour necrosis factor-α*.

**Table 6:** Changes in the expression of proteins within adipose tissue (adipocytes and cells of the stromal fraction) with age and obesity compared to young and lean, respectively.

	Proteins expressed within adipose tissue			
	Ageing	Ageing references	Obesity	Obesity references
CCR-2	↑	(170)	↑	(223)
CCR-5	↑	(170)	↑	(111, 112)
CCR-7	↓	(170)	↓	(224)
CX3CR-1	↓	(170)	↑ ← →	(225–228)
CXCR-3	↑	(170)	↑	(229)
PGE_2_	↑	(51)	↑	(230)
PPAR-γ	↓	(51)	↑	(231)

*↓, decrease; ↑, increase; ← →, little or no change or equivocal data; CCR, (C-C motif) chemokine receptor; CX3CR-1, (C-X3-C motif) chemokine receptor 1; CXCR-3, (C-X-C motif) chemokine receptor 3; PGE_2_, prostaglandin E2; PPAR-γ, peroxisome proliferator-activated receptor gamma*.

Improving our understanding of the interactions between ageing and adipose tissue dysfunction may help with the development of therapeutic and preventative strategies to improve longevity and quality of life in ageing populations. For example, with obesity, immunological dysregulation in adipose tissue is a well-established contributor to type-II diabetes and has consequently been a target for strategies such as caloric restriction, physical activity, and pharmacological manipulation. Caloric restriction imparts beneficial effects on metabolic control and blunts the production of pro-inflammatory cytokines from obese, murine adipose tissue (232, 233). Caloric restriction in older mice also delays ageing, in part due to beneficial effects on adipose tissue metabolic health and function (234). Moreover, in obese women, short- (1 month) and long-term (6 months) caloric restriction promotes an anti-inflammatory profile in abdominal, subcutaneous adipose tissue and reduces total adipose tissue macrophage content (235, 236). Physical activity can reduce systemic inflammation, targeting dysregulated chemokine and adipokine secretory patterns in mice and humans (40, 237–239). In parallel, physical activity also limits the accumulation of immune cells such as CD8+ T-cells—potentially by attenuating RANTES/CCR-5 signalling within human adipose tissue (237). Moreover, physical activity reduces neutrophil number and elastase expression, as well as shifting macrophages to an M2-like phenotype within the adipose tissue of mice fed a high-fat diet (237, 240, 241). In addition, exercise might limit, or delay immunosenescence by inducing an anti-inflammatory/anti-oxidative microenvironment within aged adipose tissue, limiting non-specific activation of T-cells and propagation of inflammation, by dampening adipose tissue-derived signals (242–244). Finally, pharmacological therapies (e.g., the PPAR-γ agonists rosiglitazone) promote M2-like macrophage repolarisation after high-fat overfeeding in mice (245). Further, PPAR signalling overlaps with the network of longevity genes, and PPAR-γ2 (adipocyte specific)-deficient mice have a considerably reduced lifespan, possibly mediated by the anti-inflammatory effects on adipose tissue macrophages (19, 20, 245). Therefore, targeting immunological dysregulation in adipose tissue might promote healthy ageing.

In conclusion, obesity is a useful model to develop our understanding of age-associated adipose tissue dysfunction. There is now an improved appreciation of how this metabolically and immunologically active tissue influences ageing and immunometabolic disease(s). The available evidence indicates that age-associated adipose tissue dysfunction occurs irrespective of changes in adipose tissue masses, and this dysfunction appears to play an active role in the pathophysiology of ageing. Moreover, the accumulation of predominantly pro-inflammatory immune cells in obese adipose tissue exacerbates the immunometabolic dysfunction of the tissue and might act as a potent stimulus for accelerating ageing in obesity. Importantly, countermeasures that target inflammation within dysfunctional adipose tissue offer promise to reduce the burden of obesity- and age-associated immunometabolic diseases.

## Author Contributions

All authors contributed equally to this work.

## Conflict of Interest Statement

The authors declare that the research was conducted in the absence of any commercial or financial relationships that could be construed as a potential conflict of interest.

## Footnotes

^1^Traditionally, macrophages have been categorised as M1 (classically activated, often thought to be pro-inflammatory) and M2 (alternatively activated, often thought to be anti-inflammatory). It is now understood that this is an oversimplification of macrophage phenotype. However, much of the available research in adipose tissue, especially in mice, has maintained the M1 and M2 categorisation. In this article, for consistency and ease of comparing studies, the terms “M1-like” and “M2-like” will be used. Within adipose tissue, it is now accepted that tissue-resident macrophages have a tissue-remodelling and pro-inflammatory phenotype, representing an intermediate population in mice and humans (i.e., cells that display an M2-like surface expression profile while demonstrating an M1-like secretory profile and function) (33, 63, 79, 170, 246, 247, 248, 249). Therefore, the role of macrophages in expanded and/or dysfunctional adipose tissue is likely to be far more complex than originally envisaged.
